# Is the sentinel lymph node biopsy more sensitive for the identification of positive lymph nodes in breast cancer than the axillary lymph node dissection?

**DOI:** 10.1186/2193-1801-2-275

**Published:** 2013-06-23

**Authors:** Ann Smeets, Emi Yoshihara, Annouschka Laenen, Anneleen Reynders, Julie Soens, Hans Wildiers, Robert Paridaens, Chantal Van Ongeval, Giuseppe Floris, Patrick Neven, Marie-Rose Christiaens

**Affiliations:** Multidisciplinary Breast Center, University Hospitals Leuven, Herestraat 49, 3000 Leuven, Belgium; Department of Oncology, KU Leuven, Surgical Oncology, University Hospitals Leuven, Leuven, Belgium; Department of Public Health and Primary Care, Interuniversity Institute for Biostatistics and Statistical Bioinformatics, KU Leuven, Leuven Belgium; Laboratory of Experimental Oncology (LEO), Department of Oncology, KU Leuven, and Department of General Medical Oncology, University Hospitals Leuven, Leuven Cancer Institute, Leuven, Belgium; Department of Imaging and Pathology, KU Leuven, Radiology, University Hospitals Leuven, Leuven, Belgium; Department of Imaging and Pathology, KU Leuven, Pathology, University Hospitals Leuven, Leuven, Belgium; Department of Oncology, KU Leuven, Gynaecological Oncology, University Hospitals Leuven, Leuven, Belgium

**Keywords:** Sentinel lymph node biopsy, Axillary lymph node dissection, Breast cancer, Lymph nodes

## Abstract

Since the routine clinical use of the sentinel lymph node (SLN) procedure, questions have been raised concerning an increase in the overall percentage of node-positive patients. The goal of our study was to compare the sensitivity of the SLN procedure and the axillary lymph node dissection (ALND) for the identification of positive lymph nodes in breast cancer.

The incidence of axillary node metastasis in SLNB and ALND specimens from patients undergoing operative treatment of a primary breast carcinoma was compared retrospectively.

Logistic regression models were used to analyze the effect of various predictors on the presence of positive lymph nodes. We constructed a multivariate model including the procedure and these predictors that have shown to be related to lymph node involvement in univariate analysis. The probability of finding positive lymph nodes was thus calculated in both groups correcting for relevant predictors of lymph node involvement.

The SLNB group included 830 patients, the ALND group 320. In a multivariate analysis, adjusting for the number of foci, tumor location in the breast, tumor size, LVI, ER, PR, tumor grade and histological subtype, the probability of finding positive lymph nodes was higher with SLNB procedure than with an ALND. However, this difference was not statistically significant (OR 0.7635; CI 0.5334-1.0930, p 0.1404).

For comparable tumors, SLNB procedure is at least as sensitive as ALND for detecting positive lymph nodes.

## Introduction

The axillary lymph node status is the most important prognostic factor in patients with early breast cancer. It is determined by patient and tumor characteristics.

To determine the axillary lymph node status, axillary lymph node dissection (ALND) has been the standard of care in patients with invasive breast cancer in order to provide correct staging of the patient and to obtain good local control. In recent years, however, sentinel lymph node (SLN) biopsy has emerged as an alternative to ALND and has become the standard treatment for axillary staging in most patients with clinically node-negative breast cancer on clinical examination, ultrasound and/or fine needle aspiration cytology (Lyman et al. 2005). The SLN procedure has been proven to be a feasible, accurate and suitable method for the staging of the axilla, while avoiding the morbidity of an ALND (Hack et al. 1999).

Since its routine clinical use, questions have been raised concerning the upstaging of a subset of node-negative patients, ranging from 5%-10% in most studies, and an increase in the overall percentage of node-positive patients due to a more detailed pathological examination of the sentinel lymph node (Tvedskov et al. 2011; Smeets & Christiaens 2005). On the other hand, the SLN procedure is associated with a false negative rate of ± 5% (Veronesi et al. 2006; Cserni et al. 2003) and thus understaging of these patients. Remarkably, axillary recurrence after a negative SLN biopsy is much lower than expected and similar to axillary recurrence after ALND (0-3%) (van der Ploeg et al. 2008; Veronesi et al. 2010; Fisher et al. 2002).

ALND is unlikely to have a therapeutic benefit for most patients with breast cancer. The value of ALND, especially in patients whose nodes are found to be tumor-free, is for accurate staging. An ALND carries the risk of understaging due to limited pathological examination of the lymph nodes, which could lead to inadequate treatment.

The goal of our study was to evaluate the sensitivity of the SLN procedure and the ALND for the identification of positive lymph nodes in breast cancer.

We hypothesized that the SLNB procedure would lead to at least equal detection rate of positive lymph nodes than an ALND when correction was performed for relevant predictors. The predictors considered were tumor size, age, number of foci, location of the tumor in the breast, tumor grade, lymphovascular invasion (LVI), histologic subtype, hormone receptor status and Her2.

## Patients and methods

### Patients

Data were obtained from retrospective review of the Multidisciplinary Breast Center (MBC) database from the University Hospitals Leuven (Leuven, Belgium). From 1 January 2007 until 31 December 2009, 1300 patients with a primary operable cT1-cT2N0 invasive breast carcinoma underwent resection of the primary tumor and axillary staging by SLNB and/or ALND. The indications for SLNB were described in institutional guidelines. In patients with a positive SLN, a completion ALND was performed. The local surgical treatment consisted of wide excision followed by radiotherapy or mastectomy with or without radiotherapy. In patients with a multifocal tumor, size and location of the largest focus were used for the analysis.

We excluded patients (i) treated for a local recurrence, (ii) with a carcinoma *in situ*, (iii) who received neo-adjuvant therapy and (iv) with primary metastatic disease.

We evaluated the association between each of the following variables and lymph node involvement: age at diagnosis, number of foci, histological tumor grade, tumor location, tumor size, histological subtype, lymphovascular invasion (LVI), ER, PR, HER-2 status and the procedure used for axillary staging (SLNB or ALND). Collection of the patients' data was approved by the local ethics committee (University Hospitals Leuven).

### Sentinel lymph node localization

The sentinel lymph node (SLN) procedure was performed by injection of a radioactive (^99m^Tc-labelled nanocolloid) tracer at the level of the tumor and Patent Blue® retroareolar. The SLNs were removed surgically using a hand-held gamma-ray detection probe.

### Examination of the lymph nodes

SLNs were routinely examined by serial sectioning. Every 300 μm 2 coupes were stained, 1 with routine haematoxylin and eosin (H&E) and 1 stained immunohistochemically using cytokeratin. Lymph nodes in an ALND were examined by H&E staining using 3 sections per node. According to published guidelines, lymph nodes from lobular breast cancers, classified as lymph node negative on H&E, were additionally stained with epithelial markers.

### Statistical methods

Logistic regression models are used to analyze the effect of various predictors on the presence or absence of positive lymph nodes. Given that women with bilateral breast tumor appear twice in the data set, generalized estimating equations (GEE) are used to account for the association between the two responses from the same person.

First, we built univariate models for a number of predictors that were expected to be related to lymph node involvement. For binary or continuous predictors we calculated the odds ratio (OR) with its 95% confidence interval and the p-value. For categorical predictors with more than two levels we performed an overall test of difference between the various levels and, in case of significance, odds ratios for the pairwise comparisons between the levels. The presence of a non-linear relationship was evaluated for the continuous variables tumor size, age, and number of foci using restricted cubic splines. In a second stage, we built a multivariate model including both procedures (SLNB or ALND) and those predictors that were related to lymph node involvement in the univariate analysis or for which such a relationship has been suggested in the literature. The probability of finding positive lymph nodes was thus calculated in both groups correcting for known predictors of lymph node involvement. Two-tailed p-values were applied and p 0.05 was considered significant.

All analyses were performed using SAS software, version 9.2 of the SAS System for Windows. Copyright © 2002 SAS Institute Inc. SAS and all other SAS Institute Inc. product or service names are registered trademarks or trademarks of SAS Institute Inc., Cary, NC, USA.

## Results

A total of 1150 patients met eligibility criteria and formed the study group; 830 patients in the SLNB group, 320 patients in the ALND group. Patient and tumor characteristics are described in Table 1. Thirty-three per cent of patients in the ALND group were lymph node positive, 28% in the SLN procedure group. The percentage of clinically node-negative patients staged with SLN biopsy increased from 63% in 2007 to 86% in 2009. Tumors in the ALND group were larger (mean tumor size 27 mm versus 18 mm) and more frequently multifocal (23% versus 8%). The other characteristics were similar for both groups.

**Table 1:** **Relationships between the procedure used for axillary staging and clinicopathological factors**

Variable	ALND (n = 320)		SLNB (n = 830)	
	n	%	n	%
Mean age (yrs)	59		58	
Mean tumor size (mm)	27		18	
Number of foci				
Unifocal	245	77	762	92
Multifocal	75	23	68	8
Histologic grade				
Grade 1	48	15	192	23
Grade 2	149	47	376	45
Grade 3	123	38	262	32
Tumor location				
Lateral	169	53	447	54
Medial	75	24	207	25
Overlapping	62	19	151	18
Retroareolar	13	4	25	3
pT				
pT1	112	35	557	67
pT2-3	208	65	273	33
pN				
pN0	214	67	601	72
pN > =1	106	33	229	28
Histologic subtype				
Ductal	251	78	694	84
Other	69	22	136	16
Lymphovascular invasion				
Present	71	22	128	15
Absent	246	77	687	83
Unknown	3	1	15	2
Estrogen receptor				
Positive	270	84	743	90
Negative	50	16	87	10
Progesteron receptor				
Positive	234	73	668	80
Negative	86	27	162	20
HER-2				
Positive	31	10	75	9
Negative	289	90	748	91

In univariate analysis, the risk of lymph node involvement was increased in the ALND group compared to that of the SLNB group. However, the difference was not significant (OR 1.2972; CI 0.9826-1.7125; p 0.0663). Furthermore, the risk was significantly related to the presence of LVI, increasing tumor size, multifocality, retroareolar or lateral tumor location in the breast, and positive hormone receptor status (ER;PR). There was no significant effect of age, tumor grade, histological subtype and HER-2 (Table 2).

**Table 2:** **Univariate analysis**

Obs Test	Odds ratio	95% CI	p-value
ALND versus SLNB	1.2972	0.9826-1.7125	0.0663
Age, years	1.0031	0.9929-1.0135	0.5497
Multifocality	1.5431	1.2549-1.8975	<0.0001
Grade (overall effect)			0.4190
grade 1 versus grade 2	0.8072	0.5729-1.1373	0.2209
grade 1 versus grade 3	0.8147	0.5674-1.1697	0.2667
grade 2 versus grade 3	1.0092	0.7566-1.3463	0.9502
Tumor location (overall effect)			0.0027
lateral versus medial	1.3955	1.0131-1.9223	0.0414
lateral versus retroareolar	0.4181	0.2161-0.8091	0.0096
medial versus retroareolar	0.2996	0.1501-0.5979	0.0006
Tumor size, mm	1.0455	1.0331-1.0580	<0.0001
LVI	7.2675	5.2014-10.1545	<0.0001
Ductal carcinoma			0.6305
Lobular carcinoma			0.4873
Estrogen receptor (negative vs positive)	0.5911	0.3824-0.9138	0.0180
Progesteron receptor (negative vs positive)	0.6335	0.4552-0.8816	0.0068
HER-2 (negative vs positve)	0.9869	0.1904-5.1146	0.9875

In a multivariate analysis, adjusting for the number of foci, tumor location in the breast, tumor size, LVI, ER, PR, tumor grade and histological subtype, the probability of finding positive lymph nodes was now higher with SLNB procedure than with an ALND (Table 3). However, once more this difference was not statistically significant (OR 0.7635; CI 0.5334-1.0930, p 0.1404).

**Table 3:** **Multivariate analysis**

Predictor	p-value
ALND versus SLNB	0.1404
Multifocality	0.0006
Tumor location	0.0064
Tumor size	<0.0001
LVI	<0.0001
ER	0.5198
PR	0.1986
Tumor grade	0.3727
Ductal carcinoma	0.7130
Lobular carcinoma	0.7765

## Discussion

According to our data, SLNB with focused immunohistopathology appears to be at least as sensitive as ALND with routine lymph node analysis.

The univariate analysis did show a higher probability of detecting positive lymph nodes with an ALND. However, this is because the ALND group consists of larger and more multifocal tumors.

Our study has a few potential limitations. First, it is a retrospective study. Nowadays, it would be unethical to randomize patients between ALND and SLN biopsy because of the morbidity associated with an ALND.

Second, in our study the difference between both procedures in detecting positive lymph nodes was not significant. It might be that the sample size in our study was too small to detect small but clinically relevant differences.

Another flaw is that we did not differentiate between macro- and micrometastases. Several studies have demonstrated that intensified pathological examination of the SLNs mainly results in detection of more micrometastases (Tvedskov et al. 2011; van der Heiden-van der Loo et al. 2006). Patients with axillary lymph nodes containing micrometastases have a higher disease recurrence and a 10%-20% lower overall survival than patients with tumor-free axillary nodes (Colleoni et al. 2005; Truong et al. 2010). Thus, more accurate staging is important, even if the increase in positive nodal status is mainly due to micrometastases.

The strength of our study is the selection of a homogeneous group of patients: we compared both techniques in patients operated on in recent years and during the same time period. Additionally, only patients with a cT1-2 N0 tumor were included in the analysis as in the period 2007–2009 only these patients were candidates for a SLN procedure. Additionally, we corrected for all known predictors of lymph node involvement.

Other studies investigating the accuracy of both techniques in detecting positive lymph nodes found a stage migration of 4-10% after the introduction of the SLN biopsy (Vanderveen et al. 2006; Meiers et al. 2013; Maaskant et al. 2009; Feinstein 1985; Giuliano et al. 1995). Most of these studies included patients from two different time periods which might imply a shift in risk factors for having lymph node metastases over time. It thus becomes more difficult to compare different time periods (Tvedskov et al. 2011; van der Heiden-van der Loo et al. 2006; Vanderveen et al. 2006; Maaskant et al. 2009). The only study comparing both techniques in patients operated on during the same time period was published by Giulianio in 1995. They concluded that the SLNB increases the accuracy of axillary staging in breast cancer and can identify significantly more patients with lymph node metastases than ALND (Giuliano et al. 1995).

In addition to the main findings, we showed that positive nodal status was mainly related to the presence of LVI, increasing tumor size and multifocality, which is in accordance to the literature (Patani et al. 2007; Viale et al. 2005; Chua et al. 2001). Moreover, we showed that negative nodal status was significantly related to medial localization of the tumor in the breast. The small number of published studies that evaluated the predictive value of tumor location in the process of lymph node involvement showed similar findings to our study (Lohrisch et al. 2000; Bevilacqua et al. 2002).

Reports on the correlation between the hormone receptor status and the presence of lymph node metastases are controversial (Viale et al. 2005; Bevilacqua et al. 2002). According to our data, patients with hormone receptor positive tumors have a higher incidence of lymph node involvement in univariate analysis but not in multivariate analysis.

The results of our study are reassuring. There is a tendency to extend the indications for SLNB to the majority of patients with clinically node-negative axilla. Based on our results, a SLN biopsy provides correct staging while avoiding the morbidity of an ALND. In spite of this, there are still surgeons with a sceptic attitude to SLNB procedure. Hopefully, our data can help to persuade them to routinely implement the SLN biopsy in the surgical treatment of patients with breast cancer.

## Conclusion

For comparable tumors, SLNB procedure is at least as sensitive as ALND for detecting positive lymph nodes.

## References

[CR1] Bevilacqua J (2002). A prospective validated model for predicting axillary node metastases based on 2,000 sentinel node procedures: the role of tumour location [corrected]. Eur J Surg Oncol.

[CR2] Chua B (2001). Frequency and predictors of axillary lymph node metastases in invasive breast cancer. ANZ J Surg.

[CR3] Colleoni M (2005). Size of breast cancer metastases in axillary lymph nodes: clinical relevance of minimal lymph node involvement. J Clin Oncol.

[CR4] Cserni G (2003). Pathological work-up of sentinel lymph nodes in breast cancer. Review of current data to be considered for the formulation of guidelines. Eur J Cancer.

[CR5] Feinstein DI (1985). Lupus anticoagulant, thrombosis, and fetal loss. N Engl J Med.

[CR6] Fisher B (2002). Twenty-five-year follow-up of a randomized trial comparing radical mastectomy, total mastectomy, and total mastectomy followed by irradiation. N Engl J Med.

[CR7] Giuliano AE (1995). Improved axillary staging of breast cancer with sentinel lymphadenectomy. Ann Surg.

[CR8] Hack TF (1999). Physical and psychological morbidity after axillary lymph node dissection for breast cancer. J Clin Oncol.

[CR9] Lohrisch C (2000). Relationship between tumor location and relapse in 6,781 women with early invasive breast cancer. J Clin Oncol.

[CR10] Lyman GH (2005). American society of clinical oncology guideline recommendations for sentinel lymph node biopsy in early-stage breast cancer. J Clin Oncol.

[CR11] Maaskant AJ (2009). Stage migration due to introduction of the sentinel node procedure: a population-based study. Breast Cancer Res Treat.

[CR12] Meiers P, Cil T, Guller U, Zuber M (2013). Sentinel lymph node biopsy in early-stage breast cancer patients: improved survival through better staging?. Langenbecks Arch Surg.

[CR13] Patani NR, Dwek MV, Douek M (2007). Predictors of axillary lymph node metastasis in breast cancer: a systematic review. Eur J Surg Oncol.

[CR14] Smeets A, Christiaens MR (2005). Implications of the sentinel lymph node procedure for local and systemic adjuvant treatment. Curr Opin Oncol.

[CR15] Truong PT (2010). Micrometastatic node-positive breast cancer: long-term outcomes and identification of high-risk subsets in a large population-based series. Ann Surg Oncol.

[CR16] Tvedskov TF (2011). Stage migration after introduction of sentinel lymph node dissection in breast cancer treatment in Denmark: a nationwide study. Eur J Cancer.

[CR17] van der Heiden-van der Loo M (2006). Introduction of sentinel node biopsy and stage migration of breast cancer. Eur J Surg Oncol.

[CR18] van der Ploeg IM (2008). Axillary and extra-axillary lymph node recurrences after a tumor-negative sentinel node biopsy for breast cancer using intralesional tracer administration. Ann Surg Oncol.

[CR19] Vanderveen KA (2006). Upstaging and improved survival of early breast cancer patients after implementation of sentinel node biopsy for axillary staging. Ann Surg Oncol.

[CR20] Veronesi U (2006). Sentinel-lymph-node biopsy as a staging procedure in breast cancer: update of a randomised controlled study. Lancet Oncol.

[CR21] Veronesi U (2010). Sentinel lymph node biopsy in breast cancer: ten-year results of a randomized controlled study. Ann Surg.

[CR22] Viale G (2005). Predicting the status of axillary sentinel lymph nodes in 4351 patients with invasive breast carcinoma treated in a single institution. Cancer.

